# Testing verbal quantifiers for social norms messages in cancer screening: evidence from an online experiment

**DOI:** 10.1186/s12889-019-6997-5

**Published:** 2019-05-29

**Authors:** Sandro T. Stoffel, Maria Goodwin, Monika Sieverding, Ivo Vlaev, Christian von Wagner

**Affiliations:** 1 Research Department of Behavioural Science and Health, University College London, Gower Street, London, WC1E 6BT UK; 2 Institute of Psychology, Heidelberg University, Heidelberg, 69117 Germany; 3 Warwick Business School, University of Warwick, Coventry, CV4 7AL UK

**Keywords:** Decision making, Social norms, Quantifiers, Nudge, Cancer screening, Online experiment

## Abstract

**Background:**

Studies have shown that presenting correct information about group norms to correct misperceptions of norms can influence health behaviours. In two online studies we investigated how different ways of communicating the current uptake of 43% of the English Bowel Scope Screening (BSS) programme affects intention among disinclined men and women.

**Methods:**

In the first study, 202 participants were asked to interpret eight quantifiers for 43% uptake (‘*few’*, ‘*many’*, *‘a considerable number’*, ‘*a large number’*, ‘*a great number’*, ‘*a lot’*, ‘*numerous’* and ‘*nearly half’*) and to indicate how misleading they perceived each of them to be. In the second study, with 1245 participants, we compared the motivational impact of two quantifiers (‘*a large number’* and ‘*nearly half’* which were associated with the highest perceived uptake (48.9%) and considered least misleading in study 1 respectively) with a control message that did not contain any information on uptake, and a message which communicated actual uptake as a proportion (43%).

**Results:**

While we found that both verbal quantifiers increased screening intentions compared with the control group (from 7.8 to 12.5%, aOR 1.72; 95%CI 1.00–2.96 in the case of ‘*a large number’* and 14.3%*,* aOR 2.02; 95%CI 1.20–3.38 for ‘*nearly half’*), simply communicating that 43% do the test, however, had no impact on intentions (9.9% vs. 7.8% aOR 1.25; 95%CI 0.73–2.16).

**Conclusion:**

Verbal quantifiers can be used to improve the perception of low uptake figures and avoid a demotivating effect.

**Electronic supplementary material:**

The online version of this article (10.1186/s12889-019-6997-5) contains supplementary material, which is available to authorized users.

## Background

Individual decision making, and behaviour is often influenced by the perception of other people’s behaviour (descriptive social norms) and what behaviour is approved by other important people and society (injunctive norms) [1, 2]. Social norms provide people with a standard behaviour for a specific situation from which they do not want to deviate [3]. Social norms can therefore be defined as rules that are understood by members of a group [4]. Various studies have shown that social norms positively influence health behaviours [5–9]. Therefore, there is growing interest in communicating normative information to encourage more people to engage in preventive health behaviours [10, 11].

While some studies have looked at the influence of social norms on cancer screening attendance or intentions [12–14] only few have tried to influence screening behaviour by communicating normative information [15–17]. Two studies have failed to encourage screening behaviour by communicating high uptake and preferences for a specific screening test, but they used relatively low social norms messages [15;16]. In Sieverding and colleagues’ experimental study with men aged 45 or older, they compared intentions following either a high (65%), low (18%) or no prevalence message [15]. They found that men in the low-prevalence group reported less intention to undergo cancer screening and were less likely to leave their name and address to receive further information about cancer screening by mail.

Similarly, a recent study by Schwartz and colleagues, that used verbal information about people’s choice of bowel cancer screening tests, such as many people, did not find any effect on intention, test preference, or uptake [16].

In a recent experimental study by von Wagner and colleagues it was shown that correcting an initial belief about colorectal cancer screening uptake upwards (i.e. stating the correct answer was initial belief plus 30%) increased screening intentions among previously screening disinclined men and women [17]. In their study, they initially asked participants to estimate how many people out of 10 they believe do the test and then provided them with a social norms messages that stated that uptake is higher than estimated or correct. Importantly, the messages used in their study were specifically designed to prove that, in principle, correcting normative beliefs increases intentions. For this purpose, they used messages that mapped on to the participants’ pre-conceived hypothesis rather than actual uptake of 43% of the English Bowel Scope Screening (BSS), which consists of an invasive flexible sigmoidoscopy test that is offered to 55 years old men and women [18]. Specifically, they communicated to disinclined participants that uptake was either what was expected, or 30 percentage points higher (e.g. 70% instead of 40%) or that uptake was 80%. They found that a social norms message stating that 80% participate in the screening programme yielded the highest impact on intention. Personalised feedback by referring to the person’s own belief did not influence this effect.

Based on the results by Sieverding and colleagues, one would expect a demotivating effect of communicating an uptake of 43% for the overall population, but as beliefs about uptake are positively correlated with own screening status [15], the information could still be motivating for non-intenders who originally believed that less than 43% participate.

Moreover, information about descriptive norms can be provided in form of concrete numbers (e.g. *43% of all eligible people do the test*) or in form of verbal quantifiers (e.g. *many eligible people do the test*). Until now, most studies that aim to address health-related intentions or behaviours use exact numbers [5–9; 15; 17]. So far only two studies have tested the use of verbal quantifiers to communicate normative behaviour in the context of cancer prevention [16; 19].

While Schwartz and colleagues did not find that communicating that many people choose the test influenced preferences or screening uptake, their study design of combining the social norms message with four additional messages does not allow us to determine whether the social norms message alone would have any effect [16].

Similarly, Zikmund-Fisher and colleagues [19] looked at whether telling women that ‘*most women*’ or ‘*a few women*’ take adjuvant chemotherapy following breast cancer surgery had a similar effect on intentions as telling them that *60%* or *5%* choose it. They found that the exact numerical norms messages about the popularity of chemotherapy had a greater effect on intentions than the less precise verbal quantifier. They conclude that verbal quantifiers are less effective due to them being less precise.

While these two studies do not suggest that verbal quantifiers are effective ways to communicate social norms, other studies advocate that their vague meaning and subjective interpretation could make them more or less motivating than exact numbers [20, 21]. In Bocklisch and colleagues’ study, participants believed that a *‘possible’* event had an average likelihood rating of 51.4 out of 100 with a standard deviation of 21.6 [20]. The large standard deviation suggests considerable variance between individuals in interpreting the verbal probability expression. Similar effects were found for frequency estimates in Wänke’s study [21].

The individual variance in verbal interpretations has primarily been assessed in terms of the problems it creates for survey research, such as the misinterpretation of Likert scales [20, 21]. Yet the vagueness of interpretations could be harnessed to influence perceptions of normative behaviour. Specifically, using verbal quantifiers for screening programmes with low uptake could mitigate the risk of demotivation as some may believe that the quantifier implies higher uptake.

### The current research

In this study, we set out to test whether verbal quantifiers could be used to increase intentions to have bowel scope screening (BSS), a test for 55 year olds offered as part of the English Bowel Cancer Screening Programme. Specifically, we wanted to compare verbal quantifiers to a precise numerical norms message and a control condition without any information about normative screening behaviour. In line with previous experimental studies [17, 22, 23], we focused on individuals who initially expressed little or no interest in BSS, to minimise ceiling and social desirability effects often associated with self-reported intention measures. [24] We also wanted to simulate a targeted intervention aimed at non-attenders who are in most need of an effective behavioural intervention. Furthermore, using only disinclined study participants, we mitigate the problem of demotivating participants as expectations about uptake is positively linked with screening behaviour [13;17]. In Sieverding and colleagues’ study, non-attenders estimated that only 28% of other men participate in the German CRC programme, whereas irregular and regular attendees estimated that between 36 and 45% do the screening test [13].

For the purpose of testing the hypothesis whether verbal quantifiers are better or worse than numerical norms in communicating low prevalence information, in terms of motivating disinclined men and women to attend BSS, we conducted two separate studies.

In Study1 participants were presented with eight quantifiers for 43%, the current uptake of BSS in England. The quantifiers are listed in Table 1 but were presented to participants in a random order Participants were asked to translate each quantifier into a proportion and then to indicate how misleading they perceived each quantifier to be after debriefing them about the true uptake of 43%.

**Table 1:** Tested verbal quantifiers for 43% uptake in Study 1

Quantifiers	
1	Few men and women who are eligible to participate do so.
2	Many men and women who are eligible to participate do so.
3	A considerable number of men and women who are eligible to participate do so.
4	A large number of men and women who are eligible to participate do so.
5	A great number of men and women who are eligible to participate do so.
6	A lot of men and women who are eligible to participate do so.
7	Numerous men and women who are eligible to participate do so.
8	Nearly half of men and women who are eligible to participate do so.

Study 2 then compared the motivational impact of two of these quantifiers with a control message that did not contain any prevalence information, and a message which communicated actual uptake as a proportion (43%). Thus, while Study 1 looked at the effect of using quantifiers on interpretation, Study 2 looked at whether descriptive norms can be used to increase intentions to participate in BSS [25]. Comparing the numerical norms message with the control condition also revealed whether uptake of 43% is perceived as demotivating or motivating in the context of BSS.

Furthermore, we also investigated in Study 2 the impact of our three normative messages on interest in reading more about the benefits and harms of the screening test. This active interest, which was demonstrated by a study participant wanting to read further information, was used as a proxy for real behaviour in line with the literature on the intention-behaviour gap [15, 26]. Additionally, we also used this question to gain a better understanding of how this nudge would facilitate or undermine people’s ability to make an informed choice about screening. Nudge type interventions such as social norms interventions have been criticized in terms of informed decision making [27–29]. As interventions should avoid being manipulative or paternalistic to enable people to make an informed choice based on knowledge of the harms and benefits of cancer screening, it is important to know whether nudges influence information seeking behaviour. As informed choice is typically measured through relevant knowledge consistent with the decision maker’s values [30], we also measure comprehension of the additional information.

We report all measures, manipulations, and exclusions in these studies. Sample size for Study 2 was calculated prior to data collection based on estimates obtained from a pilot study so that it was sufficiently powered to detect differences of at least 10% in participants choosing to do the screening test, between experimental conditions, with a power of 80% and an alpha value of 0.05 [31]. All statistical analysis was conducted with Stata/SE version 15.1 (StataCorp LP, College Station, TX).

### Study 1

The primary aim of Study 1 was to identify quantifiers that translated into the highest uptake and that were not perceived as misleading, with the view to include them into Study 2. No hypotheses were made about Study 1 due to its exploratory nature.

## Methods

### Participants

We recruited 915 men and women aged 35–54 from a survey panel (Survey Sampling International); those with a previous diagnosis of bowel cancer or who have had part of their bowel remove were excluded. Similar to previous studies, we presented eligible respondents with a description of BSS and asked them to correctly identify the test as invasive before stating their intentions to attend BSS [17;23]. For this within-person analysis, only those who stated that they would definitely (*N* = 49; 24.3%) or probably (*N* = 153; 75.7%) not do the test when invited, were included (see Additional file 1: Figure S1 for flow through Study 1). Details of the respondents’ age, ethnicity, marital status, education and employment were collected at the end of the survey (see Additional file 1: Table S1 for details about participants’ characteristics in study 1). Most people in our sample were aged between 45 and 54 (45.5%), female (54.5%), married or cohabiting (61.95), White British (82.7%), were in paid employment (75.2%) and had A-level or higher education (62.4%).

### Procedures and measures

Eligible participants were presented with eight verbal quantifiers of BSS uptake (see Table 1) in a random order and asked to translate each of them into uptake from 0 to 100%. Each participant was then asked to indicate how misleading each expression was on a scale from 0 (not misleading at all) to 100 (very misleading) when reference to the quantifier of 43% was presented. Note that before Participants were asked about how misleading they perceived the messages to be, they were first asked to translate the percentage of 43% into a proportion out of 1000 to reduce the risk of misunderstanding. We further asked participants to compare the quantifiers based on their accuracy and whether they should be used for communication to the public with the questions: ‘*Which of the following statements most accurately describes 43% participation?*’ and ‘*Which of the following statements should be used by the screening programme to describe 43% participation?’*

Participants’ numeracy and cancer health literacy were assessed by three questions adapted from Lipkus and colleagues [32] and the six questions from Dumenci and colleagues’ CHLT-6 questionnaire [33]. For both items, scores were calculated.

### Statistical analysis

As answers to the translation and misleadingness questions were not normally distributed (see Additional file 1: Figure S2 and Figure S3 for distribution of answers), we used medians as measures of central tendency and calculated confidence intervals for each quantifier using nonparametric bootstraps. Friedman and Wilcoxon signed rank tests were used to compare the quantifiers.

## Results

**Fig. 1:**
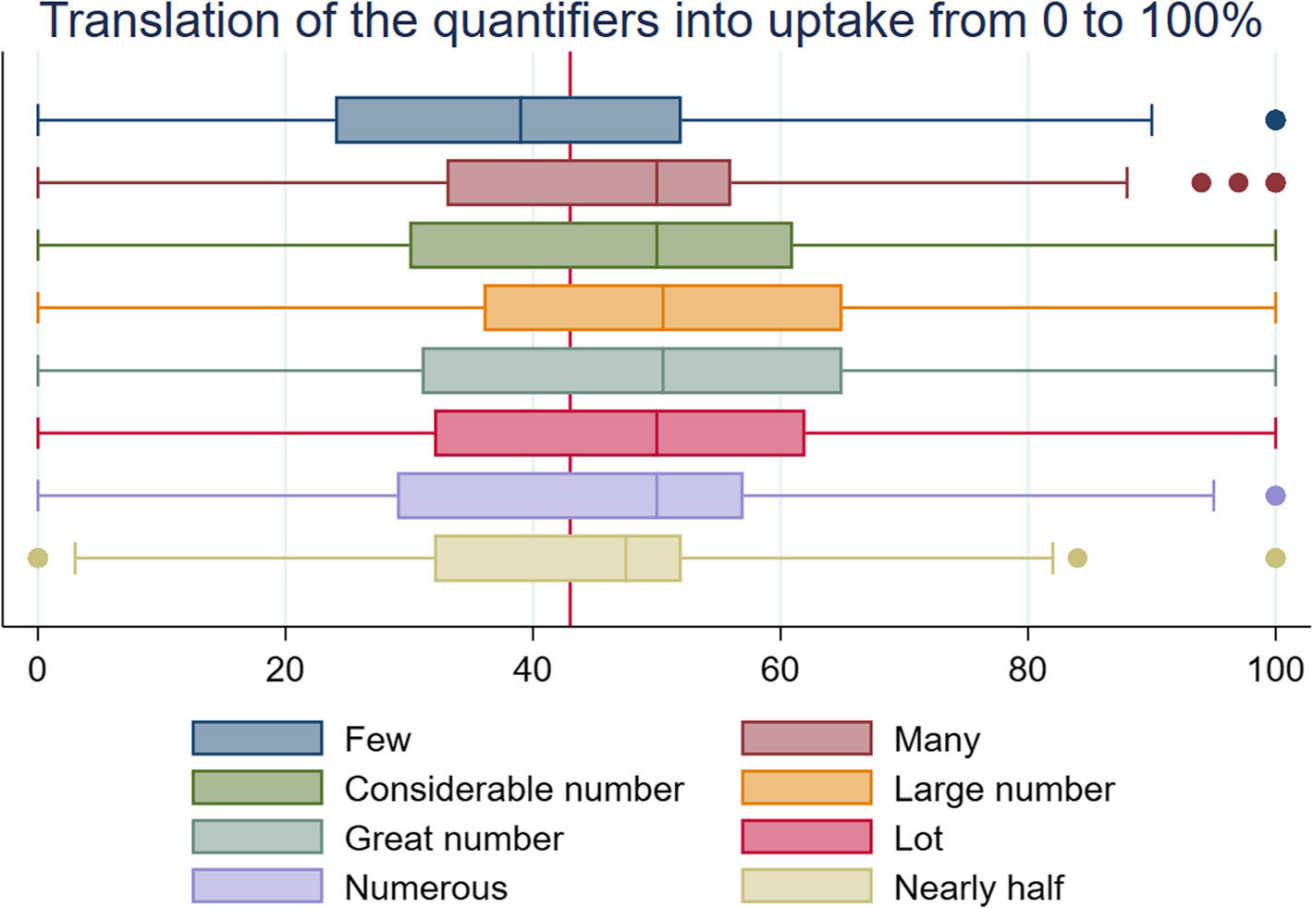
Translation of quantifier into uptake from 0 to 100% in Study 1

**Fig. 2:**
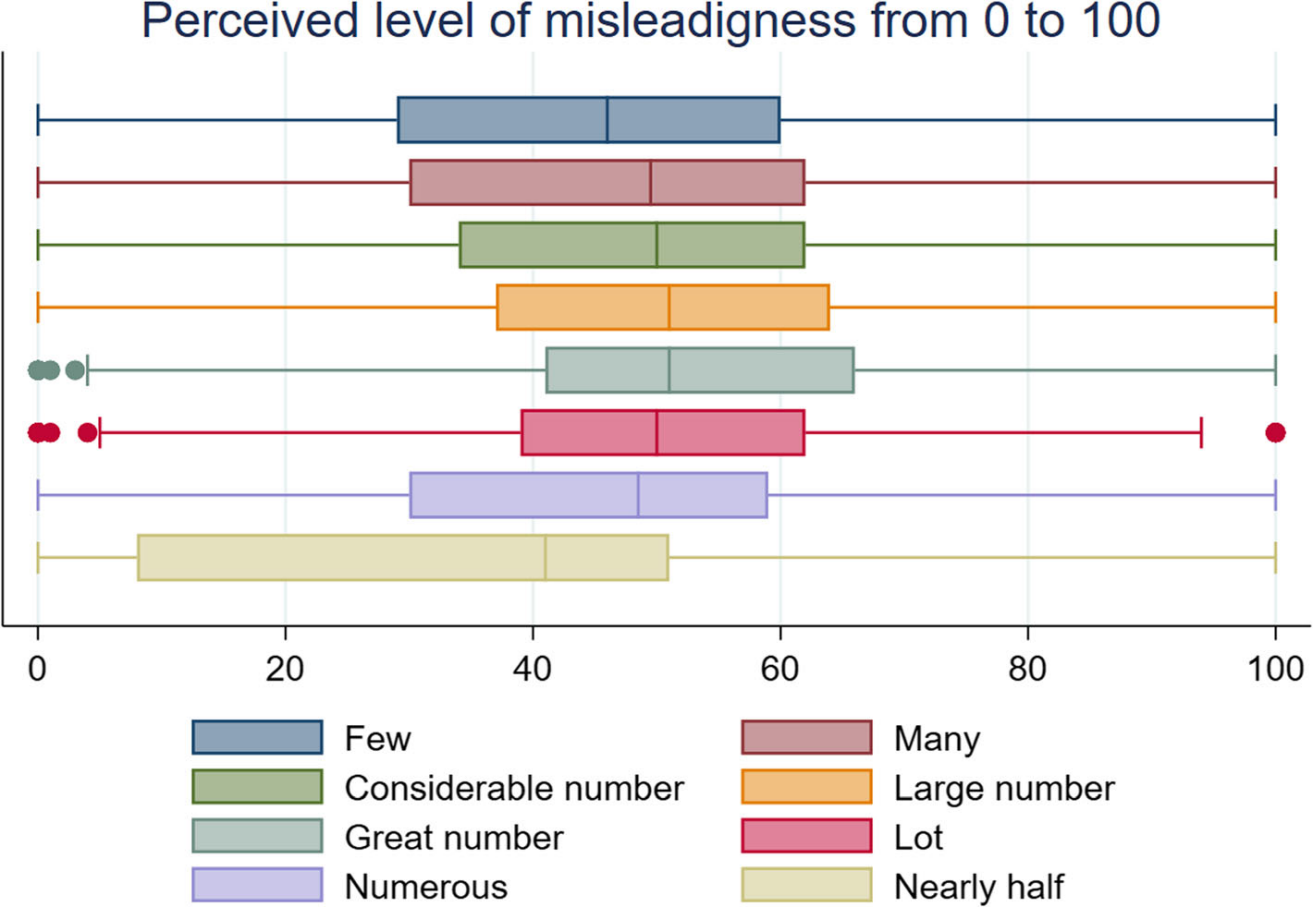
Perceived level of misperception given that quantifier refers to 43% uptake in Study 1

Table 2, as well as Figs. 1 and 2, summarises the median values of uptake and perceived misleadingness ascribed to each quantifier. A Friedman test indicated the distributions in translations of the quantifiers were significantly different between quantifiers (χ ^2^ (7) = 31.118, *p* < 0 .001*).* Wilcoxon signed rank tests showed that for all but two quantifiers (‘*numerous*’ and ‘*nearly half’*), the translations of uptake differed significantly from the true uptake value of 43%. ‘*A large number*’ and ‘*a great number*’ had the highest median translation (50.5%). All quantifiers, except for ‘*nearly half’,* were perceived as similarly misleading (χ^2^ (7) = 48.326, *p* < 0.001).

**Table 2:** Perception of the quantifiers on a scale of 0–100 in Study 1 (*N* = 202)

	Uptake translation [0;100]			Misleading [0;100]	
Quantifier	Median	(95% CI)	*p*-value✝	Median	(95% CI)
Few	39	(30.79–47.21)	0.004**	46	(42.31–49.69)
Many	50	(48.08–51.92)	0.034*	49.5	(47.44–51.56)
A considerable number	50	(48.18–51.82)	0.022*	50	(47.86–52.14)
A large number	50.5	(48.39–52.61)	0.001**	51	(49.55–52.45)
A great number	50.5	(49.02–51.97)	0.002**	51	(49.28–52.72)
A lot	50	(47.35–52.65)	0.006**	50	(49.23–50.77)
Numerous	50	(47.96–52.04)	0.267	48.5	(43.81–53.19)
Nearly half	47.5	(45.06–49.94)	0.079	41	(34.53–47.47)

✝*p*-value refers to Wilcoxon signed rank test comparing median to true uptake of 43%. *p < 0.05: **p < 0.01

*Note.* Higher translation scores indicate interpretation of higher participation, higher misperception scores indicate greater perceived deception

**Fig. 3:**
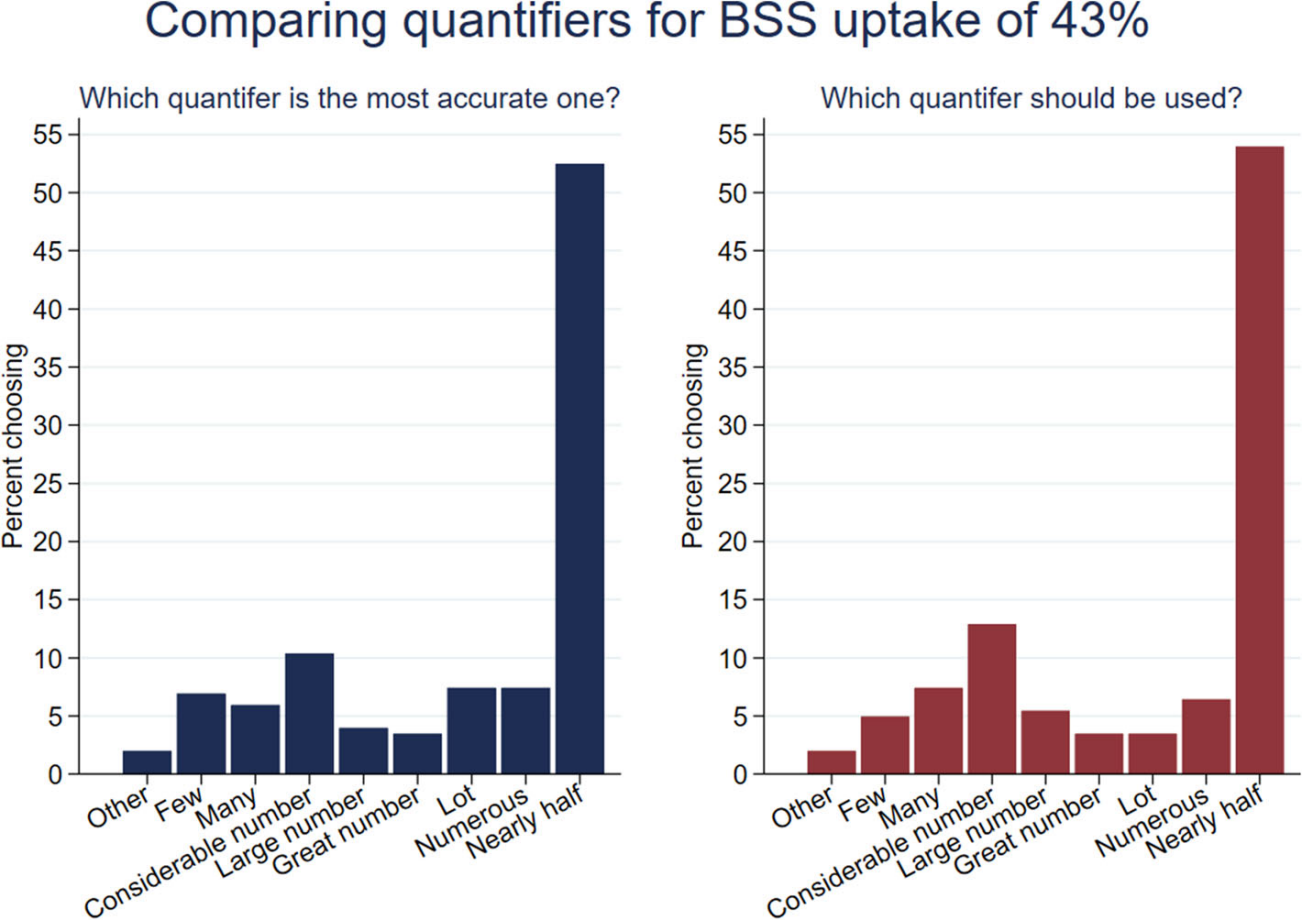
Comparing the different quantifiers for 43% screening uptake in terms of accuracy and suitability for public communication in Study 1

**Fig. 4:**
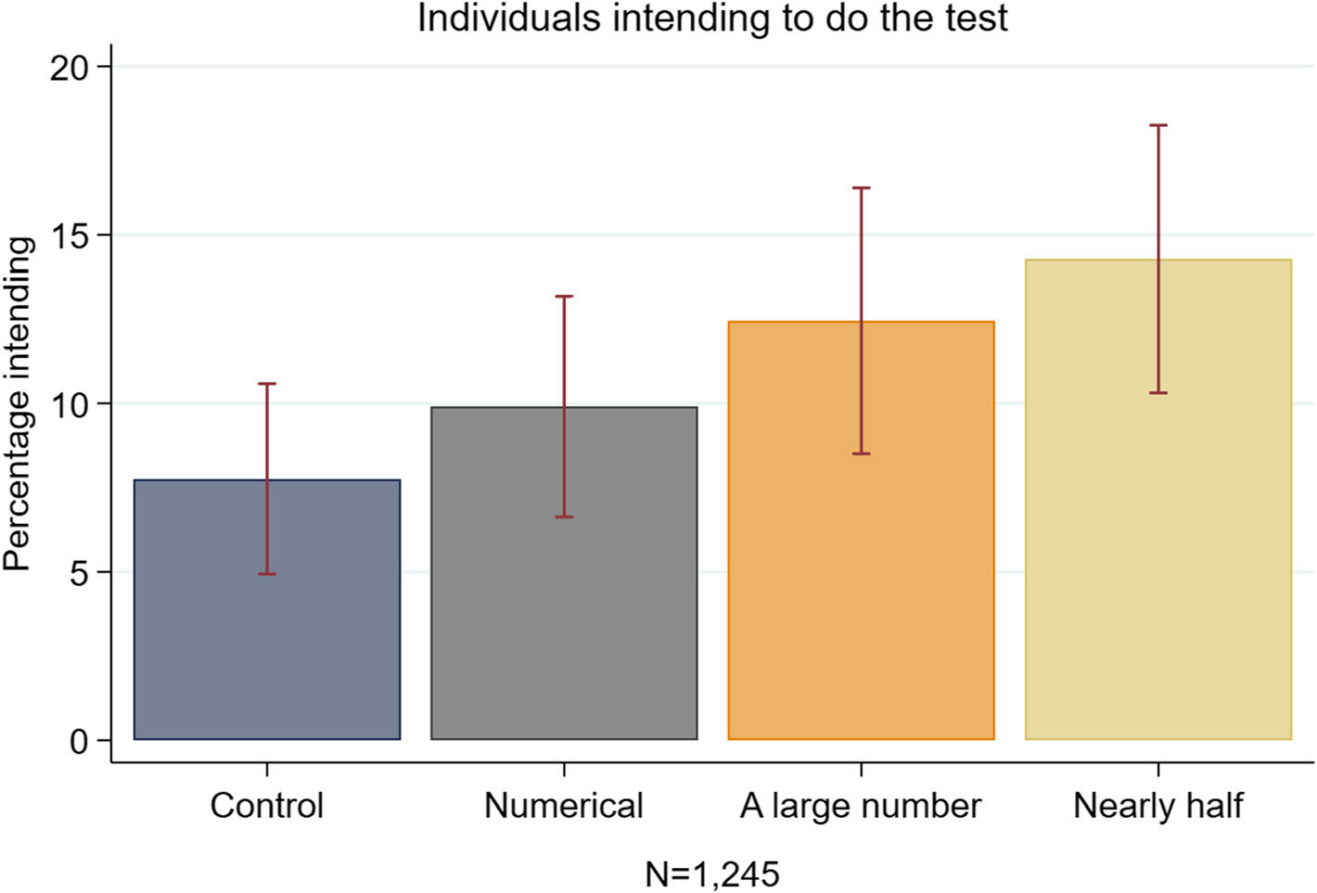
Histogram showing percentage intending (‘*yes, probably’* or ‘*yes, definitely’*) in Study 2, error bars represent 95% confidence intervals

Looking at which quantifier respondents perceived as most accurate and ideal for communication to the public, Fig. 3 reveals that ‘*nearly half*’ was the most popular choice (57.7% named it most accurate and 55.2% thought that it should be used for communication). There was a strong positive correlation between perceived accuracy and the quantifier rated best for public communication, indicating that participants thought that the public information campaigns should communicate accurate information (*r* (202) = .716, *p* < 0.001). However, the similarity between accuracy and communication ratings (Fig. 4) show the importance of accurate information. From an ethical standpoint, communicating accurate information is essential, though it is equally important to avoid adverse effects.

Results of this study confirm that individuals interpret quantifiers for screening uptake differently. Almost all quantifiers were thought to represent uptake of more than 43%. Importantly, the most popular and least misleading quantifier (‘*nearly half’)* was perceived as 43%, suggesting that using it in a normative message should not be different from communicating it as a proportion.

The next step to better understand the use of quantifiers for normative messages would be to examine whether they exert any effect on screening intentions in an experiment that features a between-subject design. To this end, Study 2 compared the most popular quantifier ‘*nearly half*’, and ‘*a large number’*, which, together with ‘*a great number’*, elicited the highest uptake but had a slightly lower misleadingness rating than the latter, with a more traditional normative message that states the proportion of people having the test. This approach allowed us to test high descriptive norms and low, but accurate descriptive norm messages.

### Study 2

The primary aim of Study 2 was to compare the effects of different normative messages on screening intentions among a group of previous non-intenders. Specifically, we compared the two normative quantifiers (‘*nearly half’* and ‘*a large number*’) with a numerical description of uptake and a message without any uptake information. Furthermore, in line with the discussion about the ethics of using normative information in cancer screening, we tested whether the messages undermine people’s ability to make an informed choice about screening and reduce the likelihood that they would decide to read further facts and figures about BSS. Finally, using comprehension checks, we looked at whether the messages affected information processing.

## Methods

### Participants

The sampling method was identical to that in Study 1 but used a different pool of participants. A total of 5484, who did not participate in Study 1, started the survey. Of the 1294 eligible respondents (see Additional file 1: Figure S4 for flow through the survey), most indicated that they would definitely not (*N =* 270; 20.9%) or probably not (*N* = 1024; 79.1%) do the test, were asked to be randomised to one of four experimental conditions with equal probability. Sociodemographic characteristics of the final sample of 1245 (96.2%) were like Study 1 and variables were balanced between the four experimental conditions (see Additional file 1: Table S2 for descriptive statistics of the study sample). Most respondents were aged 45–54 (56.5%), female (53.1%), White-British (80.0%), married or cohabiting (59.6%), in paid employment (75.8%) and had A-level or higher education (62.2%).

### Procedures and measures

Each participant received a paragraph of information about what happens during the screening test. For those in one of the three experimental conditions, an additional norms message (in bold) was added at the end of the paragraph: ‘*Currently, 43% … ’*, *‘Currently, nearly half … ’*, and *‘Currently, a large number … of men and women who are eligible to participate do so.’* Similar to Sieverding and colleagues [15], we subsequently asked participants about their intentions and whether they wished to read further facts and figures about BSS: termed active interest.

The post-exposure intention question was measured in a similar way as in the filter question, simply adding the prefix *‘Given the previous information … ’* to *“Would you take up the offer of bowel scope screening?*” and featured the same fully labelled 4-point Likert scale response options (*‘definitely not’*, *‘probably not’*, *‘probably yes’* and *‘definitely yes’*). Active interest was operationalised as the decision to read further facts and figures about BSS, rather than skipping that section. The question was adapted from a previous study and featured the response options *‘read information on next page before continuing with survey’* and *‘skip information on next page and continue with survey’* [23]. Those that opted to read the information were asked three additional multiple-choice comprehension questions to measure engagement. “*Based on what you have just read …*” was followed by (1) “*… does bowel scope screening have any physical risks?”,* (2) “*… does bowel scope screening detect all potential cancer?*” and (3) “… *how many people think that the test is painful*?”

Before debriefing, participants in all conditions were asked, based on the information they had read, how many people they thought to participate in BSS (0–100%). This question was used to measure comprehension for participants in the numerical condition, interpretation of the verbal quantifiers in the ‘*nearly half’* and *‘a large number’* conditions and beliefs about uptake in the control condition.

Finally, respondents completed the CHLT-6, numeracy skills test and demographic questions as in study 1.

### Statistical analysis

We used Chi-square tests of independence and logistic regressions adjusted for baseline intentions and sociodemographic variables to investigate the effect of the normative messages on dichotomised post-exposure intentions to participate in BSS. Intentions were reclassified (‘*yes, probably*’ and ‘*yes, definitely*’ versus ‘*probably not’* and *‘definitely not’*) due to low frequencies in some answer categories. Active interest in reading about the screening test and engagement with the information were analysed using Chi-square tests of independence and Kruskal-Wallis tests. Due to the non-normal distribution of the answers about the beliefs, understanding and comprehension of uptake, we used medians as measures of central tendency.

## Results

While Table 3 and Fig. 4 show that only the *‘nearly half’* message significantly increased screening intentions in the univariate analysis compared with the control message (14.3% vs 7.8% χ^2^(1, 649) =7.15, *p* = 0.008), and no other message significantly increased the proportion of intenders (*‘numerical’*: 9.9%, *p* = 0.326; *‘a large number’*: 12.5%, *p* = 0.051), the multivariate analysis revealed that, after adjusting for baseline intentions and sociodemographic variables, both ‘*nearly half’* (aOR 2.02, 95% CI 1.20–3.38, *p* < 0.01) and *‘a large number’* (aOR 1.72, 95% CI 1.00–2.96, *p* < 0.05) were associated with a significantly greater proportion of intenders compared to the control condition. The fFull model is included in the Supplementary file (see Additional file 1: Table S3). Note that due to the low number of study participants initially indicating that they would definitely not have the screening test when invited (*N* = 256), we could not analyse the effect of the social norms messages separately for those who answered ‘*definitely not*’ and ‘*probably not*’ at the initial intention question.

**Table 3:** Effect of normative messages on uptake intentions in Study 2

		Responders intending (%)	Unadjusted		Adjusted ^a^	
	N		Odds ratio	95% CI	Odds ratio	95% CI
Condition						
Control	348	7.8	Ref.		Ref.	
Numerical	323	9.9	1.307	0.765–2.235	1.252	0.727–2.157
Large number	273	12.5	1.691	0.993–2.880	1.721	1.002–2.955*
Nearly half	301	14.3	1.981	1.192–3.294**	2.017	1.204–3.379**
Initial intention						
Definitely not					Ref.	
Probably not					2.282	1.315–3.958**
Cancer literacy score					0.856	0.736–0.995*
*N*			1245		1245	
*R*^*2*^			0.013		0.061	

* *p* < 0.05; ** *p* < 0.01

^a^Covariates included in the adjusted models are responder’s age, gender, marital status, ethnicity, education level, employment status and numeracy skill. The full model is presented in Additional file 1: Table S3.

**Fig. 5:**
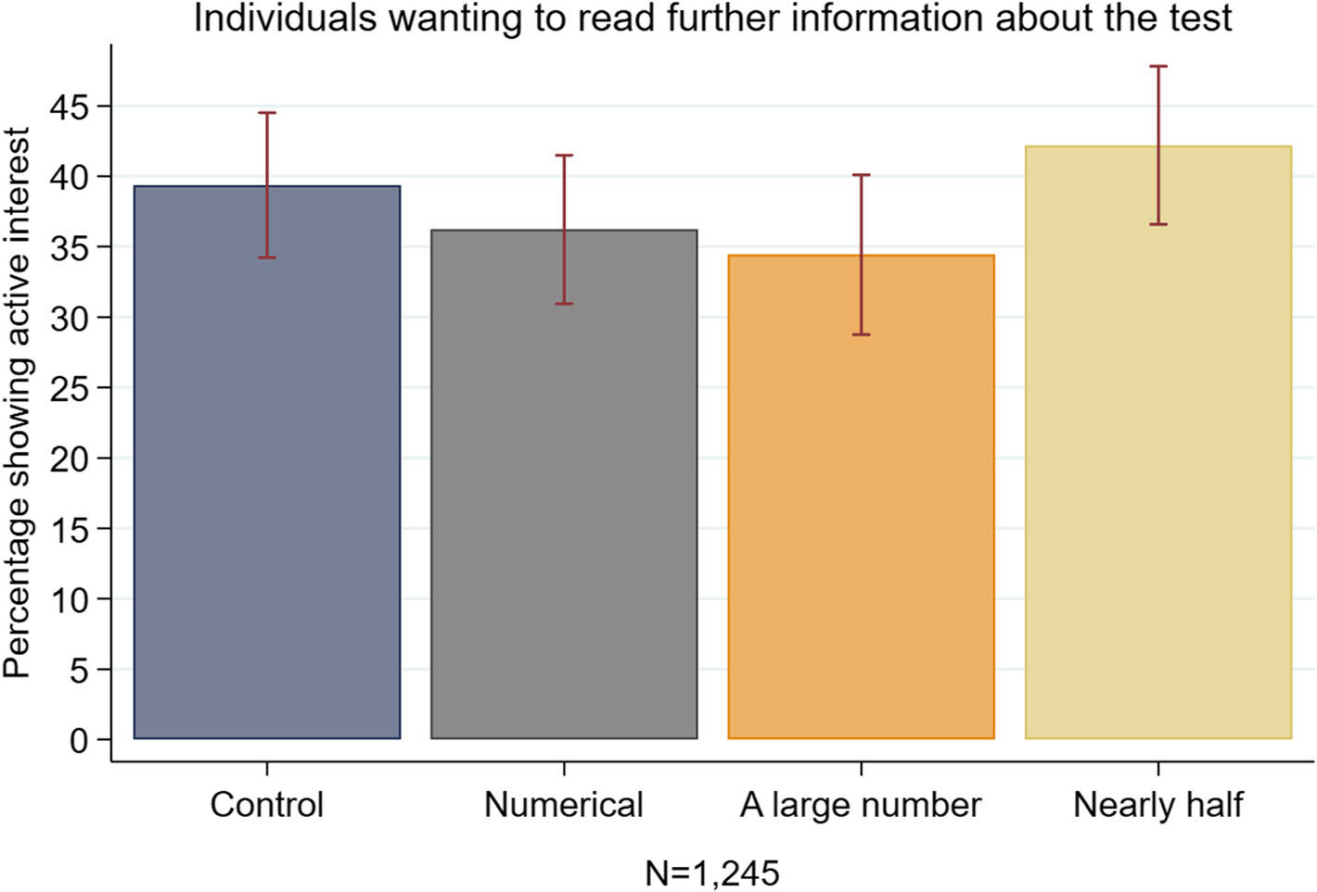
Histogram showing percentage that clicked ‘I want to read’ in Study 2, error bars represent 95% confidence intervals

Looking at whether the normative messages influenced information seeking and engagement, Table 4 and Fig. 5 reveal that, independent of experimental condition, around 38% of respondents stated that they wanted to read more (36.2–42.2%, χ^2^(3, *N* = 1245) =4.41, *p = 0.220).* Furthermore, while most participants who read the additional information about the risk and benefits of the screening test got around 2 out of 3 comprehension questions right, a Kruskal-Wallis test did not reveal any differences in BSS knowledge across the conditions (*χ*^*2*^ = 2.59, *p* = 0.274, df = 2).

**Table 4:** Effect of social norm messages on ‘active interest’ in BSS in Study 2

		Responders reading (%)	Unadjusted		Adjusted^a^	
	N		Odds ratio	95% CI	Odds ratio	95% CI
Condition						
Control	348	39.4	Ref.		Ref.	
Numerical	323	36.2	0.875	0.640–1.196	0.822	0.597–1.132
Large number	273	34.4	0.809	0.582–1.124	0.833	0.595–1.167
Nearly half	301	42.2	1.124	0.821–1.539	1.154	0.837–1.590
Initial intention						
Definitely not					Ref.	
Probably not					2.074	1.515–2.840**
Cancer literacy score					0.982	0.882–1.093
*N*			1245		1245	
*R*^*2*^			0.005		0.055	

** *p* < 0.01

^a^Covariates included in the adjusted models are responder’s age, gender, marital status, ethnicity, education level, employment status and numeracy skill. There was no difference in engagement with the additional information across the four conditions as responders who chose to read the information answered on average 1.5 questions correctly (ANOVA (3) 474 *p* = 0.533)

Additional adjusted linear regression confirmed that there was no difference across the three experimental conditions (*‘numerical’*: Beta − 0.075, 95% CI -0.289–0.139; *‘a large number’*: Beta − 0.091, 95% CI -0.318–0.137 and ‘*nearly half’*: Beta − 0.161, 95% CI -0.370–0.119; see Additional file 1: Table S4 for the full linear regression model). Thus, our normative messages did not negatively affect information seeking and processing.

The results of the Study 2 show that normative quantifiers can be used to increase interest in screening programmes with low uptake. In contrast to what we expected, the low but more accurate descriptive norm message was as motivating as the high normative quantifier. A closer look at how respondents interpreted the quantifiers only partially confirmed the findings of Study 1 (see Additional file 1: Figure S5 for distribution of answers). Respondents thought that both quantifiers referred to uptake significantly greater than 43%. As in Study 1, ‘*a large number*’ was translated into the highest uptake (*Median* = 51%, SD: 21.91); however, in contrast to Study 1 ‘*nearly half’* was translated as half (*Median* = 50, 17.76). Interestingly, both those who were provided with information about uptake in proportions and those who didn’t receive any normative message indicated at the end of the experiment that they thought uptake would be close to half (numerical condition: *Median* = 46%, SD: 17.77; control condition: *Median* = 49%, SD: 22.23). The result of the control condition is in contrast with Sieverding and colleagues’ study which suggested that non-attenders estimated that only 28% undergo CRC screening [13].

## Discussion

To our knowledge, this was the first study that compared verbal quantifiers to numerical information in the context of colorectal cancer screening. In two online surveys, we identified and tested promising verbal quantifiers for cancer screening uptake of 43%. In contrast to Zikmund-Fisher and colleagues [19], we found that communicating that ‘*nearly half’* or *‘a large number’* of men and women eligible for the test participate in the screening programme, increased intentions to do the test among previously disinclined men and women. Using an exact numerical norms message did not affect intentions. Interestingly, the quantifier ‘*nearly half*’ which was rated as least misleading and most accurate in Study 1 worked as well as the more misleading quantifier ‘*a large number*’. While this suggests that the quantifiers could be used to improve the perception of low compliance rates, the vagueness of verbal quantifiers did not appear to fully explain this, as there was no difference with regard to how participants reacted to and interpreted the two quantifiers [20]. Thus, information campaigns do not need to exaggerate the number of people who have already participated through vague and potentially misleading quantifiers but rather should correctly inform people. Furthermore, the numerical description did not decrease motivation as seen in previous experiments [15] and campaigns [34], as the communicated numerical social norms message was in line with the belief about uptake.

Importantly, we demonstrated that paraphrasing uptake using quantifiers did not negatively influence information seeking and engagement. The use of normative messages, therefore, did not seem to undermine informed decision making in the current study.

Our study has several limitations. Firstly, we only assessed intentions to participate in cancer screening and willingness to read more about the test. Therefore, the utility of verbal descriptive norms in changing screening behaviour cannot be determined. Intention does not necessarily translate to behaviours, an effect commonly referred to as the ‘intention-behaviour gap’ [26, 35]. Additional strategies may be required to build on motivational changes to aid actual screening attendance, such as implementation intentions [36]. Secondly, we only tested verbal quantifiers for one single value (43%), while a wider range of values would be needed to check the generalizability of the findings.

A further limitation was that the respondents’ first language was not controlled for. Non-native English speakers may have interpreted the verbal quantifiers differently. The issue of language may have been exacerbated by using a survey vendor that does not have a prior language skill checks. Future work should include a language check.

Moreover, the influence of perceived accuracy and credibility of normative messages on intentions and subsequent behaviours warrants further investigation. Study 1 identified a strong correlation between perceived accuracy and the quantifier rated best for public communication, echoing previous research where accuracy was considered the most important characteristic of informational messages [37].

Finally, the above suggestions exemplify how the results of the current study could be incorporated into an evidence-based leaflet or document.

## Conclusion

This study highlighted the potential of using verbal quantifiers for social norms interventions. While our systematically identified verbal quantifiers increased screening intentions among previously disinclined men and women, a traditional numerical norms message did not affect intentions. The effectiveness of using verbal quantifiers in social norms messages should be tested in other contexts and in a randomised controlled trial.

## Additional file

Additional file 1: **Figure S1.** Flow chart of participant participation through Study 1. **Figure S2.** Distributions of the translations of verbal quantifiers in Study 1; reference line depicts true uptake (43%). **Figure S3.** Distributions of misleadingness of verbal quantifiers in Study 1 (*N* = 202). **Figure S4.** Flow chart of participant participation through Study 2. **Figure S5.** Distributions of beliefs, comprehension and interpretation of social norms messages in Study 2. **Table S1.** Descriptive statistics of the study population in Study 1 (*N* = 402). **Table S2.** Descriptive statistics of the study population in Study 2 (*N* = 1245). **Table S3.** Logistic regression models on screening intentions displaying odds ratios and 95% confidence intervals (CI) – Study 2. **Table S4.** Regression models on engagement with the additional information about the screening test in Study 2. (DOCX 2744 kb)

## Abbreviations

95% CI: 95% Confidence Interval
BSS: Bowel Scope Screening
CHLT-6: 6-Item Cancer Health Literacy Test
CRC: Colorectal Cancer Screening
OR: Odds Ratio
