# Supplementary material for: Testing verbal quantifiers for social norms messages in cancer screening: evidence from an online experiment
Source: BMC Public Health. 2019 May 29;19:658. doi: 10.1186/s12889-019-6997-5 (PMC6542069; doi:10.1186/s12889-019-6997-5)

## Supporting information

**S1 Figure:** Flow chart of participant participation through Study 1.

|  | |  | | Started the survey (N=1,018) | | | |  | |  | |
| --- | --- | --- | --- | --- | --- | --- | --- | --- | --- | --- | --- |
|  | |  | | |  |  |  | | | Wrong age or diagnosed with bowel cancer (N=103) | |
|  |  |  |  |  |  |  |  | | |  |  |
|  | |  | | Eligible for the survey (N=915) | | | |  | |  | |
|  | |  | | |  |  |  | | | Drop out (N=18) | |
|  |  |  |  |  |  |  |  | | |  |  |
|  | |  | | Read the BSS description and attempted 1^st^ comprehension check (N=897) | | | |  | |  | |
|  | |  | | |  |  |  | | | Drop out (N=3) | |
|  |  |  |  |  |  |  |  | | |  |  |
|  | |  | | Correctly answered comprehension check and responded to 1^st^ intention question (N=894) | | | |  | |  | |
|  | |  |  |  |  |  |  |  |  |  | |
|  |  |  |  |  |  |  |  |  |  |  |  |
|  | **Definitely or probably not**  **(N=220)** | | | |  | | **Yes, probably or definitely**  **(N=674)** | | | |  |
|  | |  |  |  |  | |  |  |  |  | |
|  | Quota (N=202) | | | |  | | Quota (N=200) | | | |  |

**S1 Table: Descriptive statistics of the study population in Study 1 (N=402)**

|  |  | Non-intenders  (N=202) | | Intender  (N=200) | | Overall  (N=402) | | p-value* |
| --- | --- | --- | --- | --- | --- | --- | --- | --- |
| **Age** | |  |  |  |  |  |  |  |
|  | 35-44 | 110 | (54.5%) | 92 | (46.0%) | 202 | (50.2%) | 0.090 |
|  | 45-54 | 92 | (45.5%) | 108 | (54.0%) | 200 | (49.8%) |  |
| **Gender** | |  |  |  |  |  |  |  |
|  | Male | 92 | (45.5%) | 93 | (46.5%) | 185 | (46.0%) | 0.848 |
|  | Female | 110 | (54.5%) | 107 | (53.5%) | 217 | (54.0%) |  |
| **Living status** | |  |  |  |  |  |  |  |
|  | Single/div./wid.✝ | 77 | (38.1%) | 66 | (33.0%) | 143 | (35.6%) | 0.284 |
|  | Married/cohabiting | 125 | (61.9%) | 134 | (67.0%) | 259 | (64.4%) |  |
| **Ethnicity** | |  |  |  |  |  |  |  |
|  | White British | 167 | (82.7%) | 174 | (87.0%) | 341 | (84.8%) | 0.227 |
|  | Other | 35 | (17.3%) | 26 | (13.0%) | 61 | (15.2%) |  |
| **Education** | |  |  |  |  |  |  |  |
|  | No A levels | 76 | (37.6%) | 71 | (35.5%) | 147 | (36.6%) | 0.658 |
|  | A levels or higher | 126 | (62.4%) | 129 | (64.5%) | 255 | (63.4%) |  |
| **Paid employment** | |  |  |  |  |  |  |  |
|  | No | 50 | (24.8%) | 42 | (21.0%) | 92 | (22.9%) | 0.371 |
|  | Yes | 152 | (75.2%) | 158 | (79.0%) | 310 | (77.1%) |  |
| **Numeracy question (Score 0-3)** | | |  |  |  |  |  |  |
|  | Mean and SD | 2.28 | 0.85 | 2.35 | 0.78 | 2.31 | 0.82 | 0.409‡ |
| **Cancer literacy (Score 0-6)** | |  |  |  |  |  |  |  |
|  | Mean and SD | 4.67 | 1.40 | 4.94 | 1.22 | 4.80 | 1.32 | 0.047‡ |

*p-value refers to Chi-Square test of independence if not stated differently

‡p-value refers to a one-way ANOVA

✝Single, divorced or widowed

**S2 Figure:** Distributions of the translations of verbal quantifiers in Study 1; reference line depicts true uptake (43%)


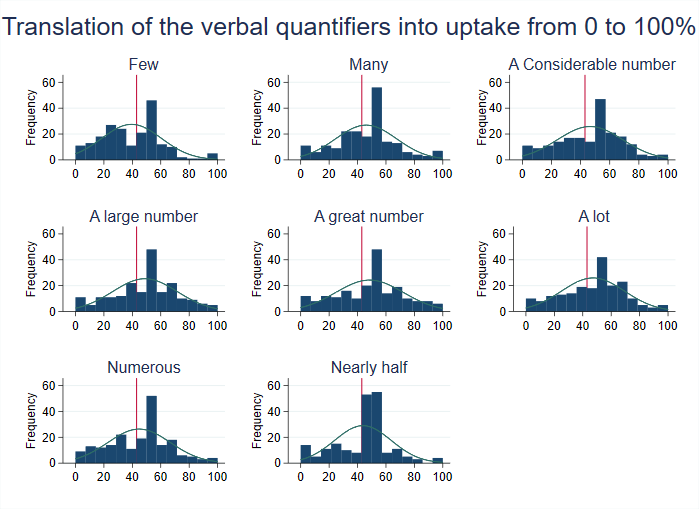


**S3 Figure.** Distributions of misleadingness of verbal quantifiers in Study 1 (N=202)


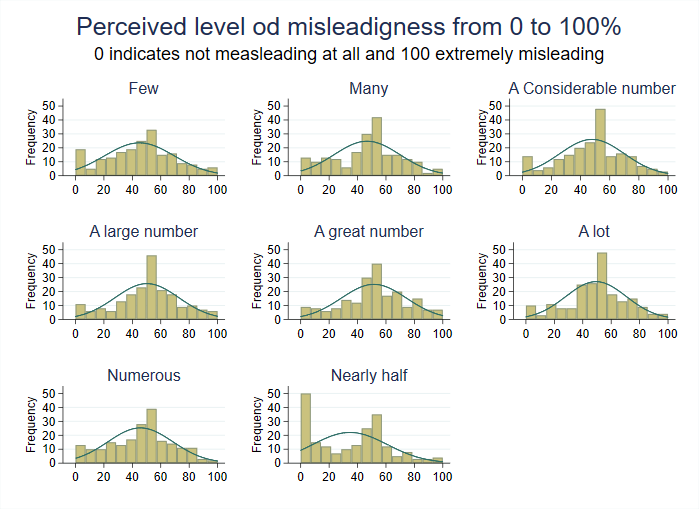


**S4 Figure:** Flow chart of participant participation through Study 2.

|  |  |  |  |  | Stated the survey (N=5,484) | | | | | | | | | |  |  |  |  |  | |
| --- | --- | --- | --- | --- | --- | --- | --- | --- | --- | --- | --- | --- | --- | --- | --- | --- | --- | --- | --- | --- |
|  |  |  |  |  |  |  |  |  |  |  |  |  |  |  |  |  |  |  |  |  |
|  |  |  |  |  |  |  |  |  |  |  |  |  |  |  |  | Diagnosed with bowel cancer/ partial bowel removal/incorrect age group (N=195) | | | |  |
|  |  |  |  |  |  |  |  |  |  |  |  |  |  |  |  |  |  |  |  |  |
|  |  |  |  |  |  |  |  |  |  |  |  |  |  |  |  |  |  |  |  |  |
|  |  |  |  |  | No bowel cancer diagnosis/ partial bowel removal and aged 35-54 years (N=5,289) | | | | | | | | | |  |  |  |  |  | |
|  |  |  |  |  |  |  |  |  |  |  |  |  |  |  |  |  |  |  |  |  |
|  |  |  |  |  |  |  |  |  |  |  |  |  |  |  |  | Drop out (N=363) | | | |  |
|  |  |  |  |  |  |  |  |  |  |  |  |  |  |  |  |  |  |  |  |  |
|  |  |  |  |  |  |  |  |  |  |  |  |  |  |  |  |  |  |  |  |  |
|  |  |  |  |  | Read the BSS description and attempted 1^st^ comprehension check (N=4,926) | | | | | | | | | |  |  |  |  |  | |
|  |  |  |  |  |  |  |  |  |  |  |  |  |  |  |  |  |  |  |  |  |
|  |  |  |  |  |  |  |  |  |  |  |  |  |  |  |  | Drop out (N=12) | | | |  |
|  |  |  |  |  |  |  |  |  |  |  |  |  |  |  |  |  |  |  |  |  |
|  |  |  |  |  |  |  |  |  |  |  |  |  |  |  |  |  |  |  |  |  |
|  |  |  |  |  | Correctly answered comprehension check and responded to 1^st^ intention question (N=4,915) | | | | | | | | | |  |  |  |  |  | |
|  |  |  |  |  |  |  |  |  |  |  |  |  |  |  |  |  |  |  |  |  |
|  |  |  |  |  |  |  |  |  |  |  |  |  |  |  |  | Probably or definitely intending to do the test (N=3,621) | | | |  |
|  |  |  |  |  |  |  |  |  |  |  |  |  |  |  |  |  |  |  |  |  |
|  |  |  |  |  |  |  |  |  |  |  |  |  |  |  |  |  |  |  |  |  |
|  |  |  |  |  | Definitely or probably not intending to do the test (N=1,293) | | | | | | | | | |  |  |  |  |  | |
|  |  |  |  |  |  |  |  |  |  |  |  |  |  |  |  |  |  |  |  |  |
|  |  |  |  |  |  |  |  |  |  |  |  |  |  |  |  |  |  |  |  |  |
| **Control** | | | |  | **Numerical** | | | |  |  | **Large number** | | | |  | **Nearly half** | | | | |
|  |  |  |  |  |  |  |  |  |  |  |  |  |  |  |  |  |  |  |  |  |
| Answered 2^nd^ intention question (N=356) | | | |  | Answered 2^nd^ intention question (N=339) | | | |  |  | Answered 2^nd^ intention question (N=282) | | | |  | Answered 2^nd^ intention question (N=311) | | | | |
|  |  |  |  |  |  |  |  |  |  |  |  |  |  |  |  |  |  |  |  |  |
| Finished survey (N=348) | | | |  | Finished survey (N=323) | | | |  |  | Finished survey (N=273) | | | |  | Finished survey (N=301) | | | | |

**S2 Table:** Descriptive statistics of the study population in Study 2 (N=1,245)

|  |  | Control  (N=348) | | Numerical  (N=323) | | Large number  (N=273) | | Nearly half  (N=301) | | Overall  (N=1,245) | | p-value* |
| --- | --- | --- | --- | --- | --- | --- | --- | --- | --- | --- | --- | --- |
| **Age** | |  |  |  |  |  |  |  |  |  |  |  |
|  | 35-44 | 152 | (43.7%) | 139 | (43.0%) | 125 | (45.8%) | 125 | (41.5%) | 541 | (43.5%) | 0.780 |
|  | 45-54 | 196 | (56.3%) | 184 | (57.0%) | 148 | (54.2%) | 176 | (58.5%) | 704 | (56.5%) |  |
| **Gender** | |  |  |  |  |  |  |  |  |  |  |  |
|  | Male | 165 | (47.4%) | 165 | (51.1%) | 115 | (42.1%) | 139 | (46.2%) | 584 | (46.9%) | 0.182 |
|  | Female | 183 | (52.6%) | 158 | (48.9%) | 158 | (57.9%) | 162 | (53.8%) | 661 | (53.1%) |  |
| **Living status** | | | |  |  |  |  |  |  |  |  |  |
|  | Single/div./wid.✝ | 144 | (41.4%) | 136 | (42.1%) | 102 | (37.4%) | 121 | (40.2%) | 503 | (40.4%) | 0.664 |
|  | Married/cohabiting | 204 | (58.6%) | 187 | (57.9%) | 171 | (62.6%) | 180 | (59.8%) | 742 | (59.6%) |  |
| **Ethnicity** | |  |  |  |  |  |  |  |  |  |  |  |
|  | White British | 276 | (79.3%) | 250 | (77.4%) | 229 | (83.9%) | 241 | (80.1%) | 996 | (80.0%) | 0.257 |
|  | Other | 72 | (20.7%) | 73 | (22.6%) | 44 | (16.1%) | 60 | (19.9%) | 249 | (20.0%) |  |
| **Education** | | | |  |  |  |  |  |  |  |  |  |
|  | No A levels | 144 | (41.4%) | 98 | (30.3%) | 104 | (38.1%) | 124 | (41.2%) | 470 | (37.8%) | 0.012 |
|  | A levels or higher | 204 | (58.6%) | 225 | (69.7%) | 169 | (61.9%) | 177 | (58.8%) | 775 | (62.2%) |  |
| **Paid employment** | | | |  |  |  |  |  |  |  |  |  |
|  | No | 83 | (23.9%) | 82 | (25.4%) | 71 | (26.0%) | 65 | (21.6%) | 301 | (24.2%) | 0.599 |
|  | Yes | 265 | (76.1%) | 241 | (74.6%) | 202 | (74.0%) | 236 | (78.4%) | 944 | (75.8%) |  |
| **Initial intentions** | |  |  |  |  |  |  |  |  |  |  |  |
|  | Definitely not | 71 | (20.4%) | 53 | (16.4%) | 64 | (23.4%) | 68 | (23.4%) | 256 | (20.7%) | 0.135 |
|  | Probably not | 277 | (79.6%) | 270 | (83.6%) | 209 | (76.6%) | 223 | (76.6%) | 979 | (79.3%) |  |
| **Numeracy question (Score 0-3)** | | | |  |  |  |  |  |  |  |  |  |
|  | Mean and SD | 2.19 | 0.90 | 2.24 | 0.87 | 2.15 | 0.88 | 2.13 | 0.92 | 2.18 | 0.89 | 0.437‡ |
| **Cancer literacy (Score 0-6)** | | | |  |  |  |  |  |  |  |  |  |
|  | Mean and SD | 4.93 | 1.22 | 4.92 | 1.27 | 4.91 | 1.24 | 4.84 | 1.22 | 4.90 | 1.24 | 0.797‡ |

*p-value refers to Chi-Square test of independence if not stated differently

‡p-value refers to a one-way ANOVA

✝Single, divorced or widowed

**S3 Table:** Logistic regression models on screening intentions displaying odds ratios and 95% confidence intervals (CI) – Study 2

|  | Model 1 | | Model 2 | | Model 3 | |
| --- | --- | --- | --- | --- | --- | --- |
|  | Odds ratio | 95% CI | Odds ratio | 95% CI | Odds ratio | 95% CI |
| **Condition** |  |  |  |  |  |  |
| Control | Ref. |  | Ref. |  | Ref. |  |
| Numerical | 1.307 | 0.765 - 2.235 | 1.277 | 0.746 - 2.186 | 1.252 | 0.727 - 2.157 |
| Large number | 1.691 | 0.993 - 2.880 | 1.730 | 1.014 - 2.950* | 1.721 | 1.002 - 2.955* |
| Nearly half | 1.981 | 1.192 - 3.294** | 2.018 | 1.212 - 3.361** | 2.017 | 1.204 - 3.379** |
| **Initial intention** |  |  |  |  |  |  |
| Definitely not |  |  | Ref. |  | Ref. |  |
| Probably not |  |  | 2.138 | 1.242 - 3.680** | 2.282 | 1.315 - 3.958** |
| **Age** |  |  |  |  |  |  |
| 35-44 years old |  |  |  |  | Ref. |  |
| 45-54 years old |  |  |  |  | 0.882 | 0.610 - 1.275 |
| **Gender** |  |  |  |  |  |  |
| Male |  |  |  |  | Ref. |  |
| Female |  |  |  |  | 0.886 | 0.609 - 1.290 |
| **Marital status** |  |  |  |  |  |  |
| Single/div./wid. |  |  |  |  | Ref. |  |
| Married/cohab. |  |  |  |  | 1.314 | 0.898 - 1.924 |
| **Ethnicity** |  |  |  |  |  |  |
| White British |  |  |  |  | Ref. |  |
| Other |  |  |  |  | 1.333 | 0.866 - 2.053 |
| **Education** |  |  |  |  |  |  |
| No A levels |  |  |  |  | Ref. |  |
| A level or higher | |  |  |  | 1.369 | 0.913 - 2.053 |
| **Paid employment** | |  |  |  |  |  |
| No |  |  |  |  | Ref. |  |
| Yes |  |  |  |  | 1.155 | 0.728 - 1.831 |
| **Numeracy score** |  |  |  |  | 0.811 | 0.649 - 1.013 |
| **Cancer literacy score** | |  |  |  | 0.856 | 0.736 - 0.995* |
| *N* | 1,245 |  | 1,245 |  | 1,245 |  |
| *R^2^* | 0.013 |  | 0.027 |  | 0.061 |  |

* *p*<0.05; ** *p*<0.01

**S4 Table:** Regression models on engagement with the additional information about the screening test in Study 2

|  |  | Median | Unadjusted | | Adjusted ✝ | |
| --- | --- | --- | --- | --- | --- | --- |
|  | N |  | Beta | 95% CI | Beta | 95% CI |
| **Condition** |  |  |  |  |  |  |
| Control | 137 | 2 out of 3 | Ref. |  | Ref. |  |
| Numerical | 117 | 2 out of 3 | -0.038 | -0.258 – 0.182 | -0.075 | -0.289 – 0.139 |
| Large number | 94 | 2 out of 3 | -0.085 | -0.319 – 0.148 | -0.091 | -0.318 – 0.137 |
| Nearly half | 127 | 2 out of 3 | -0.155 | -0.370 – 0.060 | -0.161 | -0.370 – 0.048 |
| **Initial intention** | |  |  |  |  |  |
| Definitely not |  |  |  |  | Ref. |  |
| Probably not |  |  |  |  | -0.014 | -0.174 – 0.145 |
| **Cancer literacy score** | |  |  |  | 0.050 | -0.018 – 0.119 |
| *N* |  |  | 475 |  | 475 |  |
| *R^2^* |  |  | 0.002 |  | 0.060 |  |

* *p*<0.05; ** *p*<0.01

✝ Covariates included in the adjusted models are responder’s age, gender, marital status, ethnicity, education level, employment status and numeracy skill.

**S5 Figure.** Distributions of beliefs, comprehension and interpretation of social norms messages in Study 2


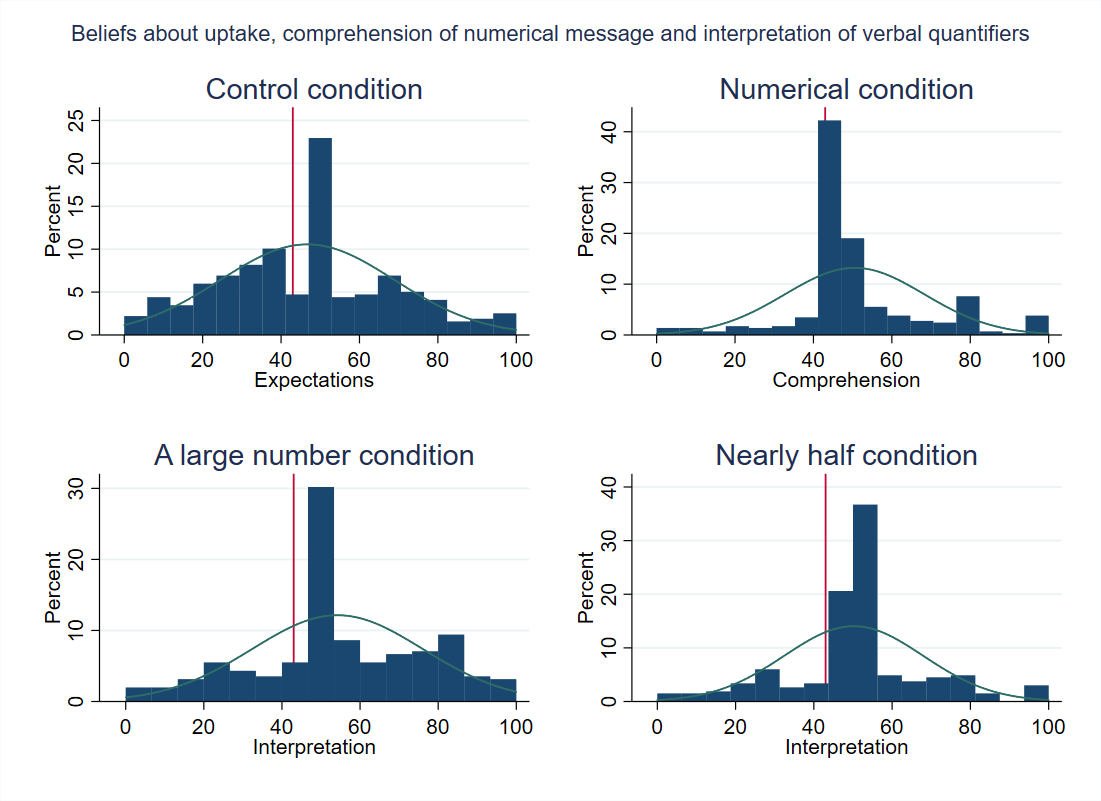

Supplement: Supplementary file 1 — Figure S1. Flow chart of participant participation through Study 1. Figure S2. Distributions of the translations of verbal quantifiers in Study 1; reference line depicts true uptake (43%). Figure S3. Distributions of misleadingness of verbal quantifiers in Study 1 (N = 202). Figure S4. Flow chart of participant participation through Study 2. Figure S5. Distributions of beliefs, comprehension and interpretation of social norms messages in Study 2. Table S1. Descriptive statistics of the study population in Study 1 (N = 402). Table S2. Descriptive statistics of the study population in Study 2 (N = 1245). Table S3. Logistic regression models on screening intentions displaying odds ratios and 95% confidence intervals (CI) – Study 2. Table S4. Regression models on engagement with the additional information about the screening test in Study 2. (DOCX 2744 kb) [file 12889_2019_6997_MOESM1_ESM.docx]
